# Echocardiogram: The GPS to GPA's Heart (Granulomatosis with Polyangiitis)

**DOI:** 10.1155/2019/7609386

**Published:** 2019-01-16

**Authors:** Manpreet K. Parmar, Mariam Alikhan, Vivien M. Hsu, Amanda Borham

**Affiliations:** Robert Wood Johnson Medical School, Piscataway Township, NJ, USA

## Abstract

Granulomatosis with polyangiitis (GPA) is a rare ANCA-associated necrotizing granulomatous vasculitis affecting small- to medium-sized vessels. Common manifestations of this disease process affect the ear, nose, throat, upper and lower airways, and kidneys. Cardiac involvement has been reported in 6–44% of patients, primarily as coronary arteritis and pericarditis. A majority of case reports of pericardial effusions in patients with GPA identify patients having constrictive pericarditis secondary to uremia. We are presenting a case of hemorrhagic pericarditis in a patient with GPA in which the underlying inflammatory vasculitis likely played the primary role in the patient's presentation. Echocardiographic abnormalities have been found in 80% of patients with GPA. Given the high mortality from cardiac involvement in patients with GPA, screening echocardiograms for this patient population may serve as a helpful tool in gauging disease severity, thereby guiding therapy to prevent serious cardiac complications, such as cardiac tamponade as presented in this case report.

## 1. Case

The patient is a 49-year-old obese Hispanic male with a past medical history of granulomatosis with polyangiitis (GPA) complicated by ESRD on hemodialysis who presented with dyspnea and chest pain. He was diagnosed with GPA in 2010 via a renal biopsy showing crescentic glomerulonephritis and treated with corticosteroids, plasmapheresis, and cyclophosphamide but had inconsistent follow-up thereafter. He repeatedly visited the ED for epistaxis and ear infections. Pathology from a 2013 arteriovenous fistula (AVF) repair showed granulomatous inflammation.

**Figure 1:**
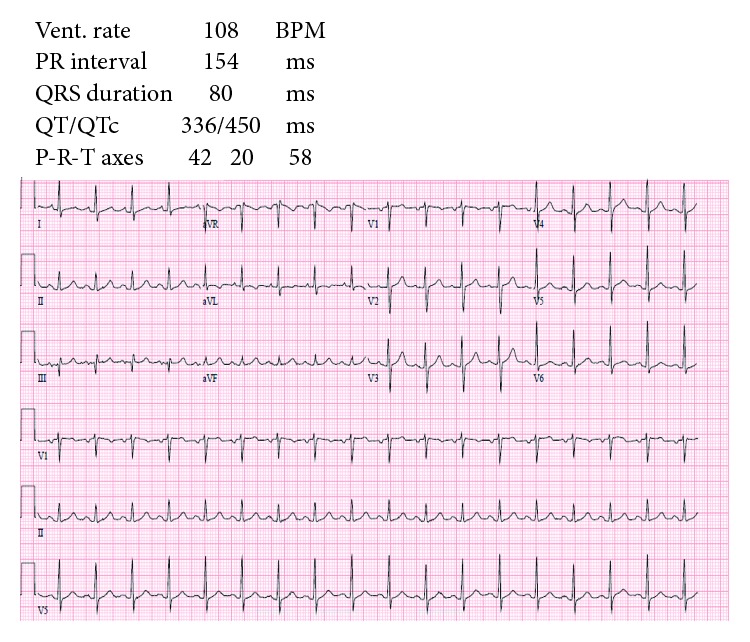
EKG showing sinus tachycardia with S wave in lead I, Q wave in lead III, and electrical alternans.

**Figure 2:**
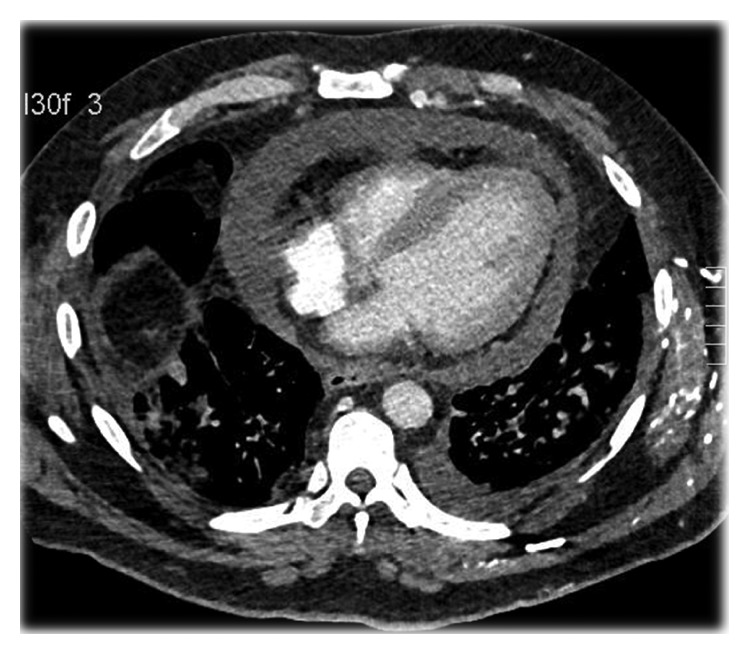
CTA chest with a moderate pericardial effusion.

**Figure 3:**
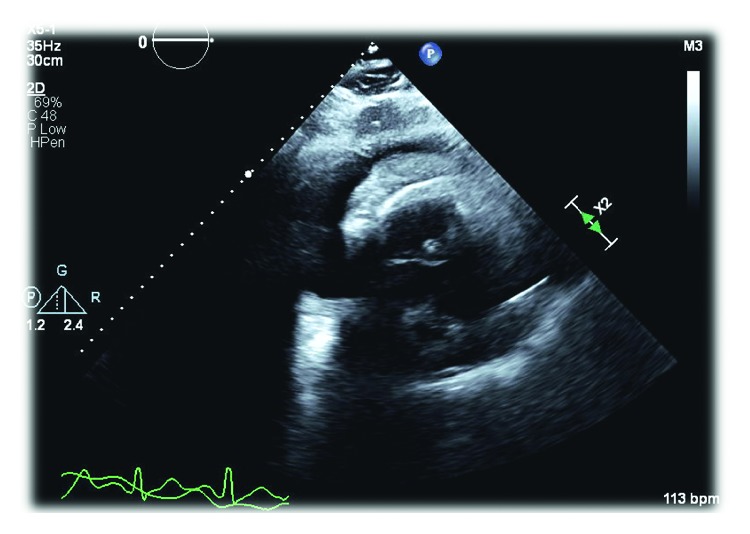
Echocardiogram showing tamponade with solid component in effusion consistent with fibropurulent exudate.

On admission, the patient was tachycardic, tachypneic, and hypoxic. Exam was notable for saddle nose deformity, distant heart sounds, jugular venous distention, and an AV fistula bruit. Lab examinations revealed normocytic anemia, elevated BUN (56 mg/dL) and Cr (12.3 mg/dL), hyperkalemia, hypophosphatemia, elevated acute phase reactants (ESR 91 and CRP 46.98), elevated *α*_1_ and *α*_2_ globulins, and increased *κ* and *λ* free light chains. Further workup showed negative c-ANCA, positive p-ANCA, elevated myeloperoxidase antibodies (>8 U), normal serine protease 3 antibodies (<0.2 U), and normal complement levels. EKG showed sinus tachycardia with S wave in lead I, Q wave in lead III, and electrical alternans (Figure 1). CTA was negative for pulmonary embolism but revealed a moderate pericardial effusion and bilateral pulmonary opacities (Figure 2). Subsequent echocardiogram was consistent with tamponade with a solid component in the effusion (Figure 3).

The patient received emergent dialysis and a pericardial window. Pericardial fluid was bloody, and pericardial tissue pathology showed acute inflammation, granulation tissue, and fibrinopurulent exudate. He was prescribed pulse dose steroids with a taper and plan for outpatient follow-up for cyclophosphamide initiation. Unfortunately, our patient was rehospitalized within a month of discharge for occlusion of his AVF and sepsis from CMV colitis and *E coli* bacteremia. He sadly passed away secondary to cardiogenic shock and hypoxic respiratory failure during this hospitalization.

## 2. Discussion

GPA is an ANCA-associated necrotizing granulomatous vasculitis affecting small- to medium-sized vessels. Several diagnostic criteria have been proposed, such as the 1990 American College of Rheumatology criteria, the revised 2012 Chapel Hill Consensus Conference criteria, and the European Medicines Agency algorithm. Hallmarks of the disease include involvement of the upper airways (70–100% of cases, including otorhinolaryngologic symptoms), lower airways, and kidneys. The ten-year survival is decreased with renal involvement from 60% to 70% to about 40% [1, 2]. Our patient had upper airway involvement from destructive sinonasal disease resulting in a saddle nose deformity along with recurrent otitis media, lower airway disease with pulmonary infiltrates, glomerulonephritis, and a positive ANCA. This patient did not demonstrate cardinal features of eosinophilic granulomatosis, such as asthma and peripheral eosinophilia.

Although GPA is primarily associated with antiproteinase 3 (PR3) antibodies, about 10–20% of patients have antimyeloperoxidase (MPO) antibodies [3]. Biopsy of an artery or perivascular area, usually obtained from the skin or the kidney, is valuable in confirming the diagnosis. Renal biopsies often show pauci-immune glomerulonephritis, which is further categorized based on histology as focal, cresentric (as in the case of our patient), sclerotic, or mixed. Treatment with immunosuppressive agents can range from methotrexate, cyclophosphamide, rituximab, glucocorticoids, azathioprine, and possibly plasma exchange depending on the severity of disease.

Cardiac involvement was first described by Wegner in 1936, and since then, it has been reported in 6–44% of patients diagnosed with GPA, with the latter attributed to those with greater disease severity. Alongside coronary arteritis, pericarditis is the most common cardiac manifestation (accounting for about 50% of all cardiac cases). Other cardiac presentations include ischemia, heart failure, valvular disorders, conduction abnormalities, and myocarditis. A majority of GPA cases with pericardial effusions and tamponade have been attributed to constrictive pericarditis secondary to uremia rather than the autoimmune process [4]. In light of the elevated disease markers, pericardial fibrin deposition, and clinical improvement with glucocorticoids, we feel that our patient fits the latter minority, with GPA as the likely culprit for his hemorrhagic pericarditis. Uremic pericarditis is associated with a marked azotemia (BUN usually > 60 mg/dL) [5] but may have also partly contributed to this patient's presentation.

Interestingly, echocardiographic abnormalities have been found in 80% of patients with GPA, but only one-third was attributed to the inflammatory disease. However, patients with cardiac involvement may be missed if asymptomatic, as in pericarditis [4]. A recent study found the prevalence of pericardial effusions to be quite low during remission, affecting only 3 of the 80 patients [6]. Given the high mortality from cardiac involvement in GPA [7, 8], early therapy is crucial for disease management. Screening echocardiograms may serve as a pivotal tool in patients with GPA to gauge disease severity, guide therapy, and prevent serious complications such as pericardial tamponade as in our patient. Fortunately, the utility of screening echocardiograms for patients with GPA has begun to gain favor [9], and we are hopeful that it will continue to become a popular tool used to augment management of this rare yet unforgiving vasculitis.
